# 2-Chloro-*N*-(2,5-dichloro­phen­yl)acetamide

**DOI:** 10.1107/S1600536809019898

**Published:** 2009-05-29

**Authors:** B. Thimme Gowda, Sabine Foro, Hiromitsu Terao, Hartmut Fuess

**Affiliations:** a Department of Chemistry, Mangalore University, Mangalagangotri 574 199, Mangalore, India; b Institute of Materials Science, Darmstadt University of Technology, Petersenstrasse 23, D-64287 Darmstadt, Germany; c Faculty of Integrated Arts and Sciences, Tokushima University, Minamijosanjima-cho, Tokushima 770-8502, Japan

## Abstract

The conformation of the N—H bond in the structure of the title compound, C_8_H_6_Cl_3_NO, is *anti* to the C=O bond. The N—H H atom shows close intra­molecular N—H⋯Cl  hydrogen bonds with both the ring Cl atom in the *ortho* position and the side-chain Cl atom. The mol­ecules crystallize in planes parallel to (221).

## Related literature

For the preparation, see: Shilpa & Gowda (2007 ▶); Pies *et al.* (1971 ▶). For our work on the effect of ring and side-chain substitutions on the solid-state geometries of aromatic amides, see: Gowda Foro & Fuess (2008 ▶); Gowda, Kožíšek *et al.* (2008 ▶); Gowda *et al.* (2009 ▶). 
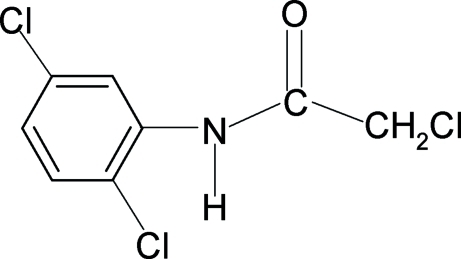


## Experimental

### 

#### Crystal data

C_8_H_6_Cl_3_NO
*M* *_r_* = 238.49
Triclinic, 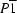

*a* = 7.492 (2) Å
*b* = 8.496 (2) Å
*c* = 8.988 (2) Å
α = 69.68 (2)°
β = 67.54 (2)°
γ = 66.67 (2)°
*V* = 472.4 (2) Å^3^
*Z* = 2
Mo *K*α radiation
μ = 0.92 mm^−1^
*T* = 299 K
0.38 × 0.28 × 0.22 mm

#### Data collection

Oxford Diffraction Xcalibur diffractometer with a Sapphire CCD detector
Absorption correction: multi-scan (*CrysAlis RED*; Oxford Diffraction, 2007 ▶) *T*_min_ = 0.720, *T*_max_ = 0.823
2735 measured reflections
1914 independent reflections
1359 reflections with *I* > 2σ(*I*)
*R*_int_ = 0.018

#### Refinement

*R*[*F* ^2^ > 2σ(*F* ^2^)] = 0.040
*wR*(*F* ^2^) = 0.098
*S* = 1.02
1914 reflections
122 parameters
H atoms treated by a mixture of independent and constrained refinement
Δρ_max_ = 0.28 e Å^−3^
Δρ_min_ = −0.31 e Å^−3^

### 

Data collection: *CrysAlis CCD* (Oxford Diffraction, 2004 ▶); cell refinement: *CrysAlis RED* (Oxford Diffraction, 2007 ▶); data reduction: *CrysAlis RED*; program(s) used to solve structure: *SHELXS97* (Sheldrick, 2008 ▶); program(s) used to refine structure: *SHELXL97* (Sheldrick, 2008 ▶); molecular graphics: *PLATON* (Spek, 2009 ▶); software used to prepare material for publication: *SHELXL97*.

## Supplementary Material

Crystal structure: contains datablocks I, global. DOI: 10.1107/S1600536809019898/bt2962sup1.cif

Structure factors: contains datablocks I. DOI: 10.1107/S1600536809019898/bt2962Isup2.hkl

Additional supplementary materials:  crystallographic information; 3D view; checkCIF report

## Figures and Tables

**Table 1:** Hydrogen-bond geometry (Å, °)

*D*—H⋯*A*	*D*—H	H⋯*A*	*D*⋯*A*	*D*—H⋯*A*
N1—H1*N*⋯Cl3	0.82 (3)	2.43 (3)	2.922 (2)	120 (2)
N1—H1*N*⋯Cl1	0.82 (3)	2.45 (3)	2.933 (2)	119 (2)

**Fig. 1.**
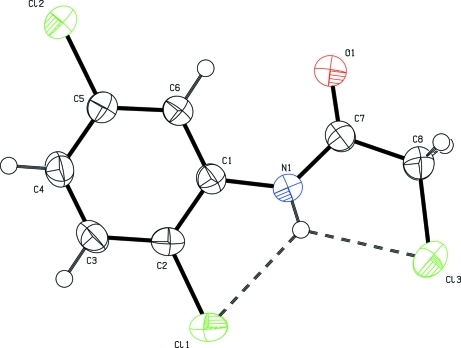
Molecular structure of the title compound, showing the atom labelling scheme. The displacement ellipsoids are drawn at the 50% probability level.

**Fig. 2.**
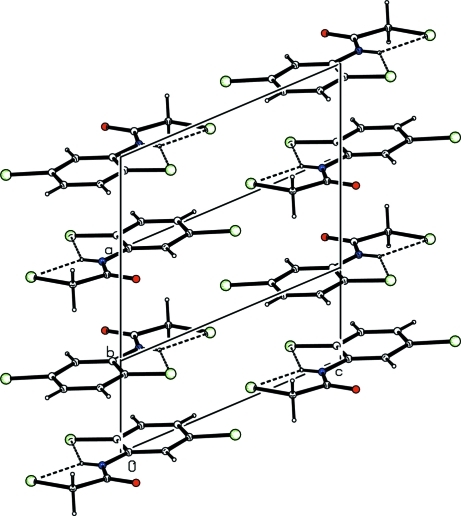
Crystal packing of the title compound with hydrogen bonding shown as dashed lines.

## supplementary crystallographic information

### Comment

As part of a study of the effect of ring and side chain substitutions on the solid state geometries of aromatic amides (Gowda Foro  & Fuess, 2008; Gowda,  Kožíšek *et al.*, 2008; Gowda *et al.*, 2009), in the present work, the structure of 2-chloro-*N*-(2,5-dichlorophenyl)acetamide (25DCPCA)(I) has been determined. The conformation of the N—H bond in the structure (Fig. 1) is *syn* to the *ortho*-chloro and *anti* to the *meta*-chloro substituents in the aromatic ring, in contrast to the *syn* conformation observed with respect to both the 2-chloro and 3-chloro groups in 2-chloro-*N*-(2,3-dichlorophenyl)acetamide (Gowda,   Kožíšek *et al.*, 2008). Furthermore, the conformation of the C=O bond is *anti* to both the N—H bond and side chain Cl atom, compared to the *anti* conformation of the C=O bond with respect to the N–H bond and *syn* with respect to the side chain Cl atom, observed in 2-chloro-*N*-(2,3-dichlorophenyl)-acetamide (Gowda Foro  & Fuess, 2008). But the conformations of the N–H bond and the side chain C–H bonds are anti to each other, while those of the ring C–Cl and the side chain C–Cl bonds are *syn* to each other. Further, the N—H H-atom shows simultaneous intramolecular hydrogen bonding with both the ring and side chain Cl atoms. The crystal packing   is shown in Fig.2 (Table 1).

### Experimental

The title compound was prepared according to the literature method (Shilpa & Gowda, 2007). The purity of the compound was checked by determining its melting point. It was characterized by recording its infrared, NMR and NQR spectra (Shilpa & Gowda, 2007; Pies *et al.*, 1971). Single crystals of the title compound used for X-ray diffraction studies were grown by a slow evaporation of its ethanolic solution at room temperature.

### Refinement

The N-bound H atom was located in difference map and its positional parameters were refined freely. The other H atoms were positioned with idealized geometry using a riding model [C—H = 0.93–0.97 Å]. All H atoms were refined with isotropic displacement parameters  set to 1.2 times of the *U*_eq_ of the parent atom.

### Crystal data

**Table:**

C_8_H_6_Cl_3_NO	*Z* = 2
*M**_r_* = 238.49	*F*(000) = 240
Triclinic, *P*1	*D*_x_ = 1.677 Mg m^−^^3^
Hall symbol: -P 1	Mo *K*α radiation, λ = 0.71073 Å
*a* = 7.492 (2) Å	Cell parameters from 698 reflections
*b* = 8.496 (2) Å	θ = 3.2–27.9°
*c* = 8.988 (2) Å	µ = 0.92 mm^−^^1^
α = 69.68 (2)°	*T* = 299 K
β = 67.54 (2)°	Prism, colourless
γ = 66.67 (2)°	0.38 × 0.28 × 0.22 mm
*V* = 472.4 (2) Å^3^	

### Data collection

**Table:**

Oxford Diffraction Xcalibur diffractometer with a Sapphire CCD detector	1914 independent reflections
Radiation source: fine-focus sealed tube	1359 reflections with *I* > 2σ(*I*)
graphite	*R*_int_ = 0.018
Rotation method data acquisition using ω and φ scans	θ_max_ = 26.4°, θ_min_ = 3.2°
Absorption correction: multi-scan (*CrysAlis RED*; Oxford Diffraction, 2007)	*h* = −8→9
*T*_min_ = 0.720, *T*_max_ = 0.823	*k* = −10→9
2735 measured reflections	*l* = −10→11

### Refinement

**Table:**

Refinement on *F*^2^	Secondary atom site location: difference Fourier map
Least-squares matrix: full	Hydrogen site location: inferred from neighbouring sites
*R*[*F*^2^ > 2σ(*F*^2^)] = 0.040	H atoms treated by a mixture of independent and constrained refinement
*wR*(*F*^2^) = 0.098	*w* = 1/[σ^2^(*F*_o_^2^) + (0.0433*P*)^2^ + 0.1195*P*] where *P* = (*F*_o_^2^ + 2*F*_c_^2^)/3
*S* = 1.02	(Δ/σ)_max_ < 0.001
1914 reflections	Δρ_max_ = 0.28 e Å^−^^3^
122 parameters	Δρ_min_ = −0.31 e Å^−^^3^
0 restraints	Extinction correction: *SHELXL97* (Sheldrick, 2008), Fc^*^=kFc[1+0.001xFc^2^λ^3^/sin(2θ)]^-1/4^
Primary atom site location: structure-invariant direct methods	Extinction coefficient: 0.043 (4)

### Special details

**Table:**

Geometry. All e.s.d.'s (except the e.s.d. in the dihedral angle between two l.s. planes) are estimated using the full covariance matrix. The cell e.s.d.'s are taken into account individually in the estimation of e.s.d.'s in distances, angles and torsion angles; correlations between e.s.d.'s in cell parameters are only used when they are defined by crystal symmetry. An approximate (isotropic) treatment of cell e.s.d.'s is used for estimating e.s.d.'s involving l.s. planes.
Refinement. Refinement of *F*^2^ against ALL reflections. The weighted *R*-factor *wR* and goodness of fit *S* are based on *F*^2^, conventional *R*-factors *R* are based on *F*, with *F* set to zero for negative *F*^2^. The threshold expression of *F*^2^ > σ(*F*^2^) is used only for calculating *R*-factors(gt) *etc*. and is not relevant to the choice of reflections for refinement. *R*-factors based on *F*^2^ are statistically about twice as large as those based on *F*, and *R*- factors based on ALL data will be even larger.

### Fractional atomic coordinates and isotropic or equivalent isotropic displacement parameters (Å^2^)

**Table:**

	*x*	*y*	*z*	*U*_iso_*/*U*_eq_	
Cl1	1.03470 (12)	0.29507 (9)	−0.22746 (8)	0.0527 (2)	
Cl2	0.72031 (12)	0.28513 (10)	0.52153 (8)	0.0594 (3)	
Cl3	0.68240 (12)	0.81217 (11)	−0.40685 (9)	0.0608 (3)	
O1	0.4705 (3)	0.7757 (2)	0.0703 (2)	0.0531 (6)	
N1	0.7131 (3)	0.5825 (3)	−0.0831 (3)	0.0375 (5)	
H1N	0.772 (4)	0.572 (3)	−0.178 (3)	0.045*	
C1	0.7880 (4)	0.4371 (3)	0.0367 (3)	0.0322 (6)	
C2	0.9420 (4)	0.2924 (3)	−0.0178 (3)	0.0335 (6)	
C3	1.0238 (4)	0.1464 (3)	0.0928 (3)	0.0389 (6)	
H3	1.1261	0.0506	0.0548	0.047*	
C4	0.9541 (4)	0.1425 (3)	0.2590 (3)	0.0395 (6)	
H4	1.0076	0.0443	0.3343	0.047*	
C5	0.8036 (4)	0.2866 (3)	0.3124 (3)	0.0376 (6)	
C6	0.7181 (4)	0.4339 (3)	0.2045 (3)	0.0370 (6)	
H6	0.6158	0.5290	0.2436	0.044*	
C7	0.5703 (4)	0.7374 (3)	−0.0617 (3)	0.0343 (6)	
C8	0.5319 (4)	0.8743 (3)	−0.2174 (3)	0.0436 (7)	
H8A	0.5540	0.9800	−0.2190	0.052*	
H8B	0.3905	0.9042	−0.2109	0.052*	

### Atomic displacement parameters (Å^2^)

**Table:**

	*U*^11^	*U*^22^	*U*^33^	*U*^12^	*U*^13^	*U*^23^
Cl1	0.0643 (5)	0.0476 (4)	0.0356 (4)	−0.0068 (4)	−0.0059 (3)	−0.0184 (3)
Cl2	0.0708 (5)	0.0553 (5)	0.0319 (4)	0.0043 (4)	−0.0182 (3)	−0.0098 (3)
Cl3	0.0635 (5)	0.0648 (5)	0.0333 (4)	−0.0041 (4)	−0.0140 (3)	−0.0049 (3)
O1	0.0577 (13)	0.0443 (11)	0.0350 (10)	0.0045 (10)	−0.0100 (10)	−0.0104 (9)
N1	0.0410 (13)	0.0352 (12)	0.0268 (11)	−0.0026 (10)	−0.0082 (10)	−0.0081 (9)
C1	0.0309 (13)	0.0310 (13)	0.0336 (13)	−0.0067 (11)	−0.0102 (10)	−0.0079 (10)
C2	0.0337 (13)	0.0358 (14)	0.0321 (13)	−0.0114 (11)	−0.0053 (11)	−0.0126 (11)
C3	0.0341 (14)	0.0336 (14)	0.0468 (16)	−0.0013 (11)	−0.0138 (12)	−0.0146 (12)
C4	0.0402 (15)	0.0333 (14)	0.0431 (15)	−0.0042 (12)	−0.0196 (12)	−0.0060 (11)
C5	0.0394 (15)	0.0384 (14)	0.0327 (13)	−0.0063 (12)	−0.0129 (11)	−0.0090 (11)
C6	0.0356 (14)	0.0358 (14)	0.0338 (13)	−0.0018 (11)	−0.0105 (11)	−0.0111 (11)
C7	0.0334 (14)	0.0313 (13)	0.0352 (14)	−0.0073 (11)	−0.0095 (11)	−0.0077 (11)
C8	0.0405 (15)	0.0421 (15)	0.0369 (15)	−0.0039 (13)	−0.0109 (12)	−0.0062 (12)

### Geometric parameters (Å, °)

**Table:**

Cl1—C2	1.737 (2)	C3—C4	1.374 (4)
Cl2—C5	1.737 (3)	C3—H3	0.9300
Cl3—C8	1.771 (3)	C4—C5	1.379 (4)
O1—C7	1.212 (3)	C4—H4	0.9300
N1—C7	1.348 (3)	C5—C6	1.383 (3)
N1—C1	1.410 (3)	C6—H6	0.9300
N1—H1N	0.82 (3)	C7—C8	1.518 (3)
C1—C6	1.389 (3)	C8—H8A	0.9700
C1—C2	1.395 (3)	C8—H8B	0.9700
C2—C3	1.382 (3)		
			
C7—N1—C1	128.8 (2)	C4—C5—C6	122.2 (2)
C7—N1—H1N	117.0 (19)	C4—C5—Cl2	119.14 (19)
C1—N1—H1N	114.0 (19)	C6—C5—Cl2	118.7 (2)
C6—C1—C2	119.1 (2)	C5—C6—C1	118.8 (2)
C6—C1—N1	122.9 (2)	C5—C6—H6	120.6
C2—C1—N1	117.9 (2)	C1—C6—H6	120.6
C3—C2—C1	120.9 (2)	O1—C7—N1	125.7 (2)
C3—C2—Cl1	119.2 (2)	O1—C7—C8	117.8 (2)
C1—C2—Cl1	119.91 (19)	N1—C7—C8	116.5 (2)
C4—C3—C2	120.1 (2)	C7—C8—Cl3	115.98 (18)
C4—C3—H3	120.0	C7—C8—H8A	108.3
C2—C3—H3	120.0	Cl3—C8—H8A	108.3
C3—C4—C5	119.0 (2)	C7—C8—H8B	108.3
C3—C4—H4	120.5	Cl3—C8—H8B	108.3
C5—C4—H4	120.5	H8A—C8—H8B	107.4
			
C7—N1—C1—C6	0.5 (4)	C3—C4—C5—Cl2	−178.1 (2)
C7—N1—C1—C2	−178.1 (3)	C4—C5—C6—C1	−0.7 (4)
C6—C1—C2—C3	0.6 (4)	Cl2—C5—C6—C1	178.5 (2)
N1—C1—C2—C3	179.3 (2)	C2—C1—C6—C5	−0.1 (4)
C6—C1—C2—Cl1	−178.90 (19)	N1—C1—C6—C5	−178.7 (2)
N1—C1—C2—Cl1	−0.2 (3)	C1—N1—C7—O1	−4.4 (5)
C1—C2—C3—C4	−0.2 (4)	C1—N1—C7—C8	175.9 (2)
Cl1—C2—C3—C4	179.3 (2)	O1—C7—C8—Cl3	179.7 (2)
C2—C3—C4—C5	−0.6 (4)	N1—C7—C8—Cl3	−0.5 (3)
C3—C4—C5—C6	1.1 (4)		

### Hydrogen-bond geometry (Å, °)

**Table:**

*D*—H···*A*	*D*—H	H···*A*	*D*···*A*	*D*—H···*A*
N1—H1N···Cl3	0.82 (3)	2.43 (3)	2.922 (2)	120 (2)
N1—H1N···Cl1	0.82 (3)	2.45 (3)	2.933 (2)	119 (2)

## References

[bb1] Gowda, B. T., Foro, S. & Fuess, H. (2008). *Acta Cryst.* E**64**, o419. 10.1107/S1600536808000408 PMC2960157 21201446

[bb2] Gowda, B. T., Foro, S., Terao, H. & Fuess, H. (2009). *Acta Cryst.* E**65**, o949. 10.1107/S1600536809011660 PMC2977650 21583993

[bb3] Gowda, B. T., Kožíšek, J., Tokarčík, M. & Fuess, H. (2008). *Acta Cryst.* E**64**, o987. 10.1107/S160053680801266X PMC2961479 21202713

[bb4] Oxford Diffraction (2004). *CrysAlis CCD* Oxford Diffraction Ltd, Köln, Germany.

[bb5] Oxford Diffraction (2007). *CrysAlis RED* Oxford Diffraction Ltd, Köln, Germany.

[bb6] Pies, W., Rager, H. & Weiss, A. (1971). *Org. Magn. Reson.* **3**, 147–176.

[bb7] Sheldrick, G. M. (2008). *Acta Cryst.* A**64**, 112–122. 10.1107/S0108767307043930 18156677

[bb8] Shilpa & Gowda, B. T. (2007). *Z. Naturforsch. Teil A*, **62**, 84–90.

[bb9] Spek, A. L. (2009). *Acta Cryst.* D**65**, 148–155. 10.1107/S090744490804362X PMC2631630 19171970

